# Belief in school meritocracy as a system-justifying tool for low status students

**DOI:** 10.3389/fpsyg.2015.01053

**Published:** 2015-07-30

**Authors:** Virginie Wiederkehr, Virginie Bonnot, Silvia Krauth-Gruber, Céline Darnon

**Affiliations:** ^1^Laboratoire de Psychologie Sociale et Cognitive, Centre National de la Recherche Scientifique, UMR 6024, Université Clermont AuvergneClermont-Ferrand, France; ^2^Laboratoire de psychologie sociale : Menaces et Société, Université Paris DescartesParis, France

**Keywords:** belief in school meritocracy, socioeconomic status, system justification, selection, school system

## Abstract

The belief that, in school, success only depends on will and hard work is widespread in Western societies despite evidence showing that several factors other than merit explain school success, including group belonging (e.g., social class, gender). In the present paper, we argue that because merit is the only track for low status students to reach upward mobility, Belief in School Meritocracy (BSM) is a particularly useful system-justifying tool to help them perceive their place in society as being deserved. Consequently, for low status students (but not high status students), this belief should be related to more general system-justifying beliefs (Study 1). Moreover, low status students should be particularly prone to endorsing this belief when their place within a system on which they strongly depend to acquire status is challenged (Study 2). In Study 1, high status (boys and high SES) were compared to low status (girls and low SES) high school students. Results indicated that BSM was related to system-justifying beliefs only for low SES students and for girls, but not for high SES students or for boys. In Study 2, university students were exposed (or not) to information about an important selection process that occurs at the university, depending on the condition. Their subjective status was assessed. Although such a confrontation reduced BSM for high subjective SES students, it tended to enhance it for low subjective SES students. Results are discussed in terms of system justification motives and the palliative function meritocratic ideology may play for low status students.

## Introduction

The belief in meritocratic ideology is the belief that, in a given system, success is an indicator of personal deservingness—namely, that the system rewards individual ability and efforts (Young, 1961; Jost et al., 2003). Meritocracy is a widespread belief in our Western society. Indeed, everyone has experienced the promotion of meritocratic messages, such as in common proverbs (e.g., “If at first we don't succeed, try, try again”; “when there is a will, there is a way”), books or movies (*The Little Engine that Could*; *The Pursuit of Happiness*), and political discourses (Democratic National Convention, “Renewing America's Promise,” 2008, see also, Ledgerwood et al., 2011; American President Investiture speech, 2012; French presidential election, 2012). These examples illustrate how Western societies focus efforts on maintaining the belief that we live “in a just world where everyone gets what he deserves—or deserves what he gets” (Lerner, 1980, p. 18).

The belief that hard work leads to success is a particularly important norm in the school environment (Duru-Bellat et al., 2009; Son Hing et al., 2011). Supporting this idea, research has shown that teachers give more value and deliver better grades to children who provide internal explanations of their behaviors, particularly when these explanations refer to efforts (Beauvois et al., 1991; Bressoux and Pansu, 2003; Dompnier et al., 2006; Dompnier and Pansu, 2010). In addition, at school, when students want to provide a positive image of themselves to their teachers, they prefer explaining their successes and failures in terms of internal characteristics (especially efforts) rather than with external explanations (Pansu et al., 2008; Dompnier and Pansu, 2010).

In spite of that, recurrent evidence shows that other factors, including social class and gender are important and consistent predictors of school performances (OECD, 2014). This evidence clearly indicates that merit is not the only determinant of school success. Why, then, should pupils and students believe in school meritocracy? Recent research underscores that meritocratic ideology can be dissociated into two separate constructs (Son Hing et al., 2011; Duru-Bellat and Tenret, 2012): *Prescriptive* meritocracy corresponds to “how people think the system should work” (i.e., desired meritocracy) whereas *descriptive* meritocracy corresponds to “how people think the system actually work”—namely, to the belief in meritocracy. In the present paper, we examine the legitimizing function of descriptive meritocracy in the context of school. We argue that belief in school meritocracy (BSM) is a system-justifying belief, and as a consequence, that people might be particularly prone to endorsing this belief, notably when merit is the only possible track to success and upward mobility.

## Belief in school meritocracy (BSM) as a system-justifying ideology

Outside of school, research has documented that people are driven to keep positive attitudes toward the actual system and the status quo (Jost et al., 2004) by preserving social hierarchies as being fair, legitimate, and justifiable through a number of system-justifying ideologies (e.g., belief in a just world, social dominance orientation). Belief in meritocracy is one of these ideologies (Jost et al., 2003; Jost and Hunyady, 2005) to the extent that it is used to legitimate existing social hierarchy and, as such, serves an ideological function (Sidanius and Pratto, 1999; Jost and Hunyady, 2005). Indeed, meritocratic ideology leads both low and high status group members to see their position in the hierarchy as fair and legitimate.

In accordance with these ideas, it has been shown that belief in meritocracy is positively associated with internal explanations of social position (Fraser and Kick, 2000; Jost, 2001), out-group favoritism for members of low status groups, and in-group favoritism for members of high status groups (Jost and Hunyady, 2005). In the same vein, Major et al. (2002) showed that the more women (low status) believed in meritocracy, the less they assigned their rejection by a man to discrimination. Moreover, when this belief was threatened, women endorsed stereotyped system-justifying explanations for men's higher status to a higher extent and were more prone to self-stereotype than when it was not (McCoy and Major, 2007). Recent research (Ledgerwood et al., 2011) has also documented that meritocratic beliefs are associated to the desire to preserve a fair and just system. Notably, when participants faced a system threat, they judged *objectively equivalent* scientific results as better in quality when they supported (vs. challenged) meritocratic beliefs. Participants also worked harder when they were told that success on the task was due to luck (rather than effort), to the extent that the task was described as useful for exploring the relationship between effort and achievement in society (Ledgerwood et al., 2011).

Taken together, these research support the idea that belief in meritocracy can serve a justifying function. However, thus far, research has exclusively focused on general meritocratic beliefs (i.e., in society). In the present paper, we focus on the more specific BSM. We argue that such a belief could be a particularly useful tool in achieving early legitimation of social inequalities, as BSM has the specificity to refer to the school system—a system particularly relevant in determining one's future position.

Indeed, several authors have identified that the educational system serves not only an educational function, but also a selection function (Darnon et al., 2009, 2012; Jury et al., 2015). The selection function of the school system refers to the fact that, in Western societies, the school system has been ascribed the role to assign pupils at various positions, which highly differ in terms of wealth, status, power, and prestige (Duru-Bellat and Tenret, 2009). As such, school grades, ranks, and diplomas are considered “merit certificates” that largely determine one's future position in society. Pupils with higher degrees are usually oriented toward high status positions while pupils with lower degrees (or no degrees), to lower status positions. Thus, because of the high stakes associated with school success and failure in determining one's future, the perceived fairness of society directly depends on the perceived fairness of the school system itself. In other words, in such a system, individuals have to believe that this selection process is fair—namely, that degrees, ranks, and grades are the pure product of their efforts and merit.

However, far from being the pure reflection of merit, school grades, ranks, and degrees also strongly reflect group belonging (e.g., being a male or a female student, being from privileged vs. unprivileged background). As an example, students from unprivileged backgrounds or those whose neither parents enrolled in higher education have fewer chances to succeed at school and in the university than upper class or continuing generation students (Robbins et al., 2004; Stephens et al., 2012; OECD, 2014), and girls and boys still strongly differ in terms of orientation and achievement (Fiske, 2012). The system-justifying power of school in producing inequalities in higher education has been recently emphasized (Bonnot and Jost, 2014; Verniers et al., in press). Indeed, each year, low social status students represent the lowest proportion of graduated students and when they obtain diplomas, they obtain lower grades than high social status students (OECD, 2014). In the same vein, only 29.7% of students in scientific field (for example, engineering schools) are women while they are 73.7% in social or literary fields (Ministère de l'Education Nationale, 2014). Thus, girls and low status students are underrepresented at university, especially in the most prestigious field (Sirin, 2005; OECD, 2014). However, admitting that school success would be determined not only by merit, but also by social group belonging (e.g., social class, gender) would question the legitimacy of those who are in a high status position and, thus, would threaten the social order. By promoting BSM, the school system is particularly efficient in justifying the social order. Indeed, by making people believe that school success is a result of individual merit, the school transforms, in some way, social class or gender differences into individual merit differences and, thus, into differences that appear to be legitimate, equitable, and fair. Such an idea is congruent with the theory of social reproduction (Bourdieu et al., 1990), according to which school promotes BSM precisely to make people accept—whatever their own status—that high status groups are more “valuable” than low status groups and, thus, deserve a higher status position within the social hierarchy. As such, BSM is a key element for maintaining the social order and rationalizing the unequal position between individuals from high vs. low status groups.

## Social status and BSM

Because of the very function of school in society, we think that, although BSM serves the interest of high status groups, members of low status groups might be particularly motivated to endorse this belief, especially when their place within the system is uncertain.

Indeed, as the system mainly serves the interest of high status groups, one could expect the members of high status groups to endorse justifying ideologies, including BSM, more than the members of low status groups (Sidanius and Pratto, 1999; Schmitt et al., 2003; Pratto et al., 2006). However, research has shown that this general assumption is not always true. In particular, according to system justification theory, there is a real “collaborative game between high and low status groups in the maintenance of status hierarchies” (Jost et al., 2003; Caricati and Lorenzi-Cioldi, 2012, p. 69). Indeed, while the meritocratic ideology is congruent with the position of members of high status groups, who are advantaged by the social hierarchies, it is conflicting for low status group members, who are disadvantaged by these hierarchies. This conflicting state explains why low status group members may also be particularly motivated to legitimize the status quo (van der Toorn et al., 2015). In fact, many researchers (Jost et al., 2003, 2004; McCoy et al., 2013) have found that system-justifying ideology, such as meritocratic beliefs, have a palliative function for members of disadvantaged groups. Indeed, as low status group members cannot restore equality, they might increase their justifying beliefs in order to reduce dissonance and preserve their group and their self-image (Jost et al., 2003). Thus, members of disadvantaged groups are also particularly likely to think that economic inequalities are legitimated and necessary and to endorse system-justifying ideologies (van der Toorn et al., 2015).

We think this might be particularly true for meritocratic beliefs. Indeed, among system-justifying ideologies (Jost and Hunyady, 2005), meritocratic ideology is specific in the sense that it promotes the idea of a possible individual upward mobility through effort. McCoy et al. (2013) demonstrated that members of low status groups might benefit from belief in meritocracy because this belief accentuates the perception of control over future results (i.e., they can exert more or less effort). They argued that the endorsement of such beliefs “may foster the perception that members of low status groups will be joining high status groups soon” (McCoy et al., 2013). Such a process allows for reconciliation between at least ego (if not group) motives with system justification motives for low status group members.

Moreover, as already mentioned, getting school degrees largely determines people's future place in society, especially for low status groups. Indeed, high status individuals benefit from several resources that are useful for increasing the chances to access to high positions in the society (e.g., financial resources, network, area of living, familiarity with the norms, and values of the system, Kraus et al., 2012; Stephens et al., 2012). As far as low status students are concerned, on the contrary, school achievement might be their only chance to achieve upward mobility and then access to high status position in society. Therefore, low status students may be particularly prone to endorsing BSM as a way to legitimate their future place in society. This should be reflected in a positive relationship with the belief that people's place in society is deserved, which should be less true for high status groups. Moreover, students from low status groups might feel especially dependent on the school system that will or will not deliver those diplomas, which are door openers for a better future. Research has shown that, when feeling highly dependent on a system, people are motivated to engage in system-justification processes, and grant more support to the status quo (e.g., Kay et al., 2009; Kay and Friesen, 2011). We thus expect that, for these students, reminding them of the selection function of university, by both reinforcing this feeling of system dependency and threatening their chances of getting higher status, would bolster their BSM. Consequently, BSM might be even enhanced when their standing within a system, on which they strongly depend for acquiring status, is particularly uncertain (due to the severe selection process). As far as students from advantaged groups (e.g., male students, high SES students) are concerned, their status advantage should protect them, to a certain extent, from feeling dependent on the school system. Indeed, even if they are not “rewarded” by the school system, they still have more chances to get ahead in life and preserve their higher status. Thus, they should admit more easily that merit is not the only determinant of success.

## Overview and hypotheses

The present article tests the hypotheses that low status students might be particularly prone to relying on BSM to justify their future place in society. Consequently, BSM should be positively linked with general beliefs in a just society, especially for low status individuals. In the first study, the relationship between general system-justification beliefs and BSM will be observed among high (i.e., boys, high SES) vs. low status (girls, low SES) high school students. In Study 2, students' place within the system will either be challenged and the system dependency reinforced, or not, depending on the condition, by making salient the selection process students experience at the university. We expect that, for low status students, BSM would be related to the endorsement of system-justifying ideology (meritocratic beliefs at the societal level; Study 1) and enhanced as a response to threat (i.e., when students face a selection process). This should not be true for high status students.

## Study 1

### Method

#### Participants

Two hundred fifty-one high school students from two schools were asked to complete the questionnaire (147 girls, 102 boys, 2 unknown; *M*_*age*_ = 15.03, *SD* = 0.31). They were in their first year of senior high school. At the time the two studies were run, no approval was needed in France to conduct research on human subjects. Data were collected in accordance to the “American Psychological Association's ethical principles of psychologists and code of conduct.” Participants were informed that questionnaires were anonymous. They could refuse to participate or withdraw from participation at any time. The agreements of the directors of each senior high school as well as parental consents were required to participate to the study. Eight students were excluded (five because they were repeaters and three because they missed to report on it), leaving 242 participants (145 girls, 97 boys) for the analyses.^1^ Controlling for high school did not change any of the results, so high school was not retained in the final model.

#### Material

##### Socio-demographic information

Several information was gathered at the end of the questionnaire, including gender of participants and their fathers' and mothers' highest school degree. As suggested by several authors (for a review, see Kraus and Stephens, 2012), parental level of education was used as an indicator of students' social class. Students whose parents had a high school degree (i.e., *baccalauréat*) or less were categorized as low SES students (*N* = 107), students whose parents had a higher level of education were categorized as high SES students (*N* = 116).

##### System justification scale

We used a French translated version from Wakslak et al. (2011) adaptation of Kay and Jost's (2003) scale for a high school population. We added a ninth item to tap the meritocracy dimension of the scale (see below). Participants answered using a 6-point scale, ranging from (1) not at all agree to (6) completely agree. A PCA with varimax rotation revealed three factors with eigenvalues superior to 1 (accounting for 62.85% of the total variance; *KM0* = 0.74, χ^2^[36] = 549.63, *p* < 0.001). The first factor contained the first six items of the scale and *reflected belief in a just French society* (e.g., “Everyone in France has a fair shot at wealth and happiness”). The second factor reflected the *belief in a meritocratic society* (two items, “In France, people generally have what they deserve”; “People's place in society largely depends on their motivation to succeed”). The last factor only comprised a reversed item (i.e., “France is getting worse every year”), so it was removed from the analyses. As both dimensions correspond to system justification and as the alpha of the complete scale is good, a score of “belief in a meritocratic and just society” was created (α = 0.75, *M* = 3.25, *SD* = 0.74).

##### Belief in school meritocracy (BSM)

Pupils were then asked to judge whether success and failure at school could be explained by various factors, using 6-point scales. BMS, our variable of interest, was measured with two items, namely, explanation of school success in terms of effort, and explanation of school failure in terms of lack of effort, *r*_(238)_ = 0.35, *p* < 0.001, *M* = 5.25, *SD* = 0.76. The others were filler items. There was no significant difference in endorsement of BSM according to gender (*p* = 0.67), but a marginal effect of SES, with low SES students endorsing BSM to a lower extent (*M* = 5.16, *SD* = 0.89) than high SES students (*M* = 5.34, *SD* = 0.60, *p* < 0.10).

### Results

#### Link between BSM and belief in a meritocratic and just society according to pupils' gender

A first regression included BSM (centered variable), students' gender (coded +0.5 for girls and −0.5 for boys), their interaction, as well as students' SES to adjust for its effect (coded −0.5 for low SES and +0.5 for high SES). System-justifying beliefs were regressed on this model. This model accounted for 5% of the variance, *F*_(4, 221)_ = 3.83, *p* = 0.005.

**Figure 1:**
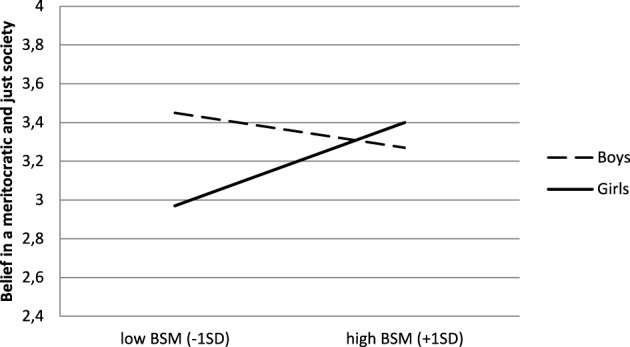
**Belief in a meritocratic and just society as a function of BSM and high school students' gender (Study 1)**.

Results indicate that boys tend to endorse System-justifying beliefs (*M* = 3.34, *SD* = 0.74) more highly than girls (*M* = 3.19, *SD* = 0.75), *b* = −0.17, *t*_(221)_ = −1.73, *p* = 0.08, η^2^_*p*_ = 0.01. There was a significant main effect of SES, as described in the following section, but no main effect of BSM (*b* = 0.06*, t* < 1). Moreover, as can be seen in Figure 1, the relationship between BSM and belief in a meritocratic and just society differed depending on students' gender, *b* = 0.30, *t*_(221)_ = 2.33, *p* = 0.02, η^2^_*p*_ = 0.02. For boys, a simple slope analysis revealed that there was no significant relationship between the variables, *b* = −0.09, *t* < 1. For girls, however, the more they explained school achievement with efforts, the more they endorsed a belief in a meritocratic and just society, *b* = 0.21, *t*_(221)_ = 2.49, *p* = 0.01.

#### Link between BSM and belief in a meritocratic and just society according to pupils' SES

The second regression used BSM, students' SES and their interactions, as well as gender to adjust for its effect, as predictors. When system justification score was used as a criterion, the model accounted for 4% of the variance, *F*_(4, 221)_ = 3.22, *p* = 0.01.

**Figure 2:**
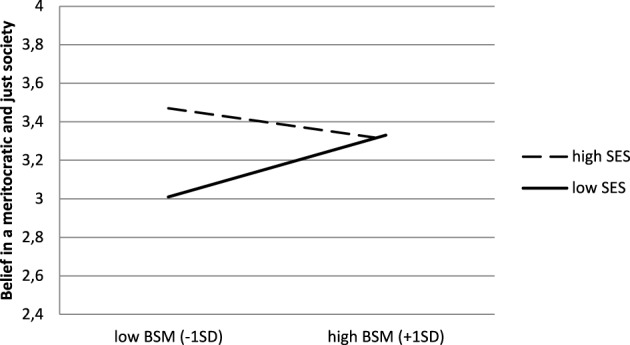
**Belief in a meritocratic and just society as a function of BSM and high school students' SES (Study 1)**.

Results were basically the same (see Figure 2). High SES students (*M* = 3.37, *SD* = 0.73) endorsed system-justifying beliefs to a higher extent than low SES students (*M* = 3.13, *SD* = 0.75), *b* = 0.22, *t*_(221)_ = 2.28, *p* = 0.02, η^2^_*p*_ = 0.02. Again there was no main effect of BSM (*b* = 0.04, *t* < 1). The interaction was marginally significant, *b* = −0.24, *t*_(221)_ = −1.75, *p* = 0.08, η^2^_*p*_ = 0.01, and simple slope analyses revealed that, although there was no significant link for high SES students, *b* = 0.10, *t* < 1, for low SES students, the more they endorsed BSM, the more they believed in a meritocratic and just society, *b* = 0.34, *t*_(221)_ = 4.21, *p* < 0.001.

### Discussion

High school students tend to endorse system-justifying beliefs to a higher extent when they come from a high status group (boys and high SES) than when coming from low status groups (girls and low SES students). However, in accordance with our hypothesis, members of low status groups seem to readily connect system-justifying beliefs at the school level (i.e., BSM) and at the societal level (i.e., belief in a meritocratic society), something that high status group members do not seem to do in the present study. Doing this might well serve a need to believe that, if they work well at school, they might acquire status later on in society, as that might be the only chance they get. If they do not succeed to climb the ladder, they will have only them to blame (i.e., their lack of efforts at school). Thus, for them, relying on BSM might be especially important to keep on believing that they actually control their future achievement in life.

The results of the first study support the idea that high endorsement of BSM may be, for girls or low SES students, a way to maintain the perception that the system is fair. In the next study, the conditions under which BSM is enhanced for low status groups are examined. Indeed, Study 1 suggests that BSM allows low status individuals to believe that the system is fair and legitimate and that they may reach a higher status position within such a system—namely, upward mobility is possible for them. Thus, BSM should be particularly high when individuals' place within the system is threatened and when they feel highly dependent on the system (Kay et al., 2009). To test this hypothesis, in Study 2, university students are examined. Indeed, the selection process is particularly salient at the university level (Darnon et al., 2009). Depending on the condition, students are either reminded of the important selection process that occurs at the university or told that the main goal of university is to allow everybody to succeed. Previous research has shown that low status students' performance decreases when the selection process of university is made salient (Jury et al., 2015) or when assessment practices focus on selection (Smeding et al., 2013). Moreover, the school system grants them (or not) a chance to achieve upward mobility, and they are consequently particularly dependent on it, all the more so as the selection process is made salient. Thus, low status students should be particularly prone to endorsing BSM when their place within the system is challenged—namely, when they are reminded of the fact that an important selection process occurs within the system.

In Study 2, participants were led to read a text that either made salient the selection process that occurs at the university or the idea that everybody can succeed. Two selection conditions were constructed: a “past selection” condition and a “future selection” condition. Participants were in their second year of study. In both selection conditions, the small percentage of selected students at University after, respectively, the first year (“past selection”) or the third year of studies (“future selection”) was reminded. On the one hand, the students of the “past selection” condition have passed through the harsh selection process, which may reinforce their beliefs in their own deservingness. On the other hand, in both selection conditions, participants were reminded of the important selection process at University and as such, both should threaten the place low SES students occupy within the University system. Thus, we think that both selection conditions should increase low SES students' reliance on BSM compared to the “success for all” condition.

Moreover, as recommended by Rubin et al. (2014), in Study 2, a subjective measure of SES was used. Indeed, unlike objective social status, subjective social status is highly context dependent. As an example, people from the “objective” middle class socioeconomic background can assess themselves as belonging to lower social class when they are in an elite university context (Kraus and Stephens, 2012). The imprecision of the “objective” measure of SES could perhaps explain why, in Study 1, the effects involving the SES variable did not reach the conventional level of significance. Thus, relying on students' self-perceptions rather than on the characteristics of their parents can convey a more accurate picture of their subjective experience of status within the university system. A final goal of Study 2 is to improve the quality of the BSM measure to ensure increased validity. To that end, a multi-item scale to measure BSM was constructed. As in Study 1, the new items focus on both success and failure at school. However, in this version, some items refer to success and failure in term of grades whereas others directly contain references on deservingness and one of them is a reverse item (*cf*. infra).

## Study 2

### Method

#### Participants

Participants were 126 second-year psychology students (19 males; 107 females; *M*_*age*_ = 20.77; *SD* = 3.22). As in Study 1, participants were informed that their answers were anonymous and that they could refuse to participate or withdraw from participation at any time during the experiment. Personal consent was required to participate to the experiment. As the measure of BSM explicitly referred to the perception of the French school system, only French nationality students were kept in the analyses; this selection resulted in a loss of seven participants. Three more participants did not respond to the subjective SES measure. The final sample comprised 116 participants (15 boys; 101 girls; *M*_*age*_ = 20.67; *SD* = 3.15). In this study, the number of male participants was too low to compare them to women. Thus, only subjective SES was used as a status variable.

#### Procedure

Participants received a booklet containing the experimental induction and the BSM measure. They were randomly assigned to one of the three experimental groups: the “past selection” group (*N* =39), the “future selection” group (*N* = 38), and the “success for all” group (*N* = 39). First, in the three experimental groups, participants read a sentence about the importance of students' success at the university. In the “success for all” condition, this sentence was followed by a neutral description of the university's administrative organization (“…University is organized into several pedagogical instances, composed of a director, teachers, staff and students, elected by their peers…”). In contrast, in both past and future selection conditions, this first sentence about the importance of students' success at the university was challenged. Indeed, the selection process that occurs at university was made salient. The text also emphasized the small percentage of selected students (27%) after, respectively, the end of the first year (“past selection”) or the end of the second year of studies (“future selection”). As participants were in their second year of study, these inductions introduced either an upstream selection process or a downstream selection process (Sommet et al., 2013). More precisely, in the “past selection” (“*future selection*”) condition, participants read: “In Psychology, more than two out of three students fail to pass the first year (*the second year*) of their studies. In 2008, for example, only 27% of the students who enrolled in the first year (*second year*) of psychology succeed in their exam and then access to the second (*third*) year of psychology. This percentage illustrates the important selection process that operates after the first (*second*) year. Second year (*third year*) students have managed to make it through this important selection process.”

### Measures

#### Belief in school meritocracy (BSM)

In the first study, participants were asked to report whether they believed that school successes and failures are explained by efforts (and a lack of efforts). As previously mentioned, one of the goals of Study 2 was to create a more subtle multi-item scale measuring BSM. Thus, drawing from the items of existing questionnaires, including the questionnaire on the Perception of Inequalities and Social Justice Survey (AVS, ISSP, PISJ), International Social Survey Program (Forsé and Parodi, 2011), and Preference for the Merit Principle Scale (Davey et al., 1999), a new scale was constructed. As we focused on descriptive meritocracy, the instruction of our scale was as follow: “We ask you to indicate to what extent you think each item corresponds to the reality of school today.” The eight items are presented in Appendix. One item was a reverse item. The scale demonstrated good internal reliability (α = 0.77; *M* = 3.73; *SD* = 0.76).

#### Subjective SES scale

As previously mentioned, a measure of students' subjective SES was used to address students' status. We used the 10-rung scale from Adler et al. (2000). Students were asked to “Think of this ladder as representing where people stand in our society. At the top of the ladder are the people who are the best off, those who have the most money, most education, and best jobs. At the bottom are the people who are the worst off, those who have the least money, least education, and worst jobs or no job” (Adler et al., 2000). As in the original procedure, students were then asked to put an “X” on the rung they thought representing their family position in society. The more students perceived themselves to occupy a high SES position, the closer their responses approached 10 (*M* = 5.62; *SD* = 1.49).

### Results

We hypothesize that, as both selection inductions should threaten the place low SES students have in the system, both should increase low SES students' reliance on BSM compared to the “success for all” condition. Thus, we expected the two selection conditions (past and future) to differ from the “success for all” condition. To test our hypothesis, the variance was divided into two orthogonal contrasts: The contrast of interest compared the two selection conditions (coded +1 each) to the “success for all” condition (coded-2). The orthogonal contrast compared the two selection conditions (coded −1 for “future selection,” +1 for “past selection,” and 0 for “success for all” conditions). The regression analysis included the two contrasts, subjective SES (centered), and their interactions.

**Figure 3:**
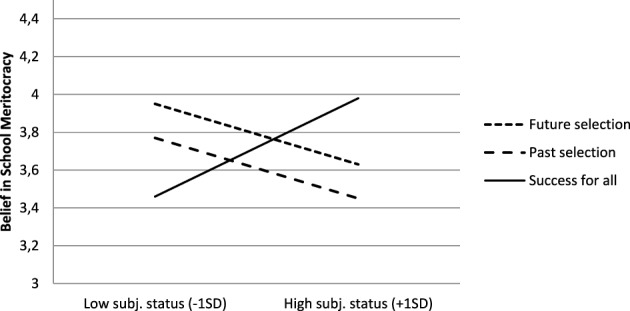
**Belief in school meritocracy (BSM) as a function of experimental condition and subjective SES (Study 2)**.

As expected, the interaction between subjective SES and the contrast of interest was significant, *b* = −0.001, *t*_(115)_ = −2.81, *p* = 0.006, η^2^_*p*_ = 0.07 (see Figure 3). The results showed that, for high subjective status students, the presence of selection information led to a lower endorsement of BSM than the “success for all” condition but the reverse occurred for low subjective status students. In simple effects analyses, we tested the effect of the contrast of interest (namely, the comparison between the two “selection conditions” and the “success for all” condition) at two different levels of the subjective SES scale (+1SD above the mean and −1SD below the mean). These analyses indicated that the contrast of interest was significant and negative for high subjective SES students, *b* = −0.16, *t*_(115)_ = 2.11, *p* = 0.04, η^2^_*p*_ = 0.08 (*M* = 3.45 for “past selection”; *M* = 3.63 for “future selection”; *M* = 3.98 for “success for all” condition). It was marginal, in the reverse direction for low subjective SES students, *b* = 0.14, η^2^_*p*_ = 0.06 (*M* = 3.77 for “past selection”; *M* = 3.95, for “future selection”; *M* = 3.46 for “success for all” condition). No other effect reached significance: all *ts* ≤ 1, *ps* > 0.32. Notably, the orthogonal contrast was not significant, *t* = 1, ns, indicating that the two selection condition did not differ from each other.

### Discussion

In Study 2, participants were confronted, or not, depending on the condition, with threatening selection information, making salient the high failure rate at the university. The results confirmed that, as expected, the effects of this information depended on participants' subjective SES. In line with the hypotheses, for students who perceived themselves to be of a low SES, this selection information increased their adherence to BSM. Interestingly, the reverse occurred for high status students, who seemed to endorse BSM to a greater extent when they read that the university policy goal was to achieve success for all students. We interpret this effect as resulting from the threat that this information might convey to them: If all students succeed, then their high status position in society is not secured anymore. Endorsing BSM might be a way for them to face this threat and bolster the status quo.

One could argue that students of the “past selection” may be particularly prone to endorse BSM because they have passed through the selection process, and thus, they want to increase their own deservingness. However, the very similar results in both selection conditions support the idea that what is determinant in the relation between social status and BSM is the salience of the selection process, and not personal past achievement or perception of own deservingness.

## General discussion

In the present paper, BSM is envisioned as a system-justifying tool allowing the preservation of groups' status hierarchies later on in life (Jost et al., 2003; Jost and Hunyady, 2005). In particular, we argue that this ideology serves a rationalizing function for low status groups who might rely on it to accept more readily the place they will have in society as being deserved.

Two studies tested this role by first looking at the relationship between BSM through pupils' explanations of school success and failure in terms of efforts and their beliefs about meritocracy in society at large (Study 1) and then by looking at the conditions under which the endorsement of BSM is increased among low status students (Study 2). Although low status pupils connect their explanations of school success (and failures) in terms of efforts to the belief that people get what they deserve in society, this connection is weaker for high status pupils. We believe that, unlike low status students for whom having a diploma is particularly important to climb the ladder, for high status students, having a diploma matters less for determining their future status. This issue is well exemplified by a participant's statement in a research interview (Brinbaum et al., 2007, p. 109): “*Someone with no diploma today still has less chance to get better along than someone else, in particular when one is not coming from a favorable background, of course. Since if ‘you are born with a silver spoon in the mouth,’ you should go well because you have parents who introduce you everywhere, because you have relations and money.”* We suspect that BSM may fulfill a palliative function for low status students to deal with their uncertain future position in the social hierarchy. Moreover, Study 2 shows that, contrary to high status students, for low status university students, reminding them of the harsh selection process operating at university leads them to paradoxically endorse BSM even more. Indeed, this particularly severe selection renders uncertain their probability of achieving upward mobility and emphasizes how dependent they are on the school system. Consequently, selection increased low SES students' reliance on BSM. Thus, taken together, the results of the two present studies document in a complementary way how BSM may serve a justifying function for low status students and help them maintaining the perception of the system as being fair and as a system in which success is possible for everybody.

Several limitations to this research should be noted. First, given that the interaction between BSM and SES did not reach the conventional level of significance we acknowledge that future studies should involve a larger set of participants in order to ascertain these links. Other limitations concern the correlational nature of Study 1 and the use of self-report measures. For these two last reasons, causality cannot be established and the possibility that a third factor may explain the relation observed cannot be excluded. For example, the difference between low and high status groups in Study 1 might reflect the fact that low and high status children do not receive equivalent parental education. Then, the measure may reflect what the participants were told about merit in school and society, rather than what they really believe. A more direct test of the implications of believing in school meritocracy for explaining one's future place in society is needed, for instance by looking at how a situation that experimentally enhances BSM might be related to larger-scale system-justifying beliefs and behaviors (e.g., salary expectations in the future). As such, we would ascertain that bolstering an ideology specific to one particular system (BSM in the school system) is used, albeit differently by high and low status group members, to justify a larger-scale system (social system) and employment. Second, we argue that endorsing BSM is a useful system-justifying tool because it increases people's perceived control over their future (McCoy et al., 2013). However, in this research, we lack empirical facts to help determine which of these factors (i.e., threat for upward mobility, system-dependency, decreased perceived control), a combination, or even an accumulation of factors explain why the selection function enhances BSM for low status students. Future research should test these hypotheses in a study that includes scales measuring these concepts in addition to the BSM scale and tests these outcomes as potential mediators.

As mentioned earlier in this manuscript, although BSM has sometimes been discussed as an important ideology of the school system (Bourdieu et al., 1990), few studies have examined what makes students endorse (or not) this belief, particularly in the school context. In this sense, we believe that the present results offer interesting perspectives for future research. Notably, they underscore that—beyond the knowledge of the existence of other predictors of school success, unrelated to merit (e.g., social class, gender)—individuals may be particularly reluctant to admit that school meritocracy does not exist if they are in a lower status position. This point also calls into question the consequences of such a belief among high and low status students. Indeed, on the one hand, one could argue that, as BSM restores low status students' sense of control over their future and their perception of society as fair, BSM could have a positive impact on low status students' achievement. On the other hand, as BSM may make people internalize their position in the school system (Jost and Hunyady, 2005) and as lower status pupils and students usually perform more poorly than higher status individuals at school, BSM might threaten the perception low status students have of their ability to succeed within the system and, consequently, might reduce their achievement. Future research should examine these two possibilities.

### Conflict of interest statement

The authors declare that the research was conducted in the absence of any commercial or financial relationships that could be construed as a potential conflict of interest.

## Footnotes

^1^The data presented in this section come from a series of questionnaires designed to investigate the influence of various ideologies on self-perceived competence, autobiographical memories, and possible selves of high school students. We report here only the results based on the first two measures appearing in the questionnaire, namely system justification measure and explanations for school achievement.

## Appendix

### Belief in school meritocracy (BSM) scale (study 2)

1. At school, when there is a will, there is a way,
2. Everyone has the same chances to succeed at school,
3. To succeed at school, one only has to work hard,
4. At school, students who obtain poor grades are those who have not worked enough,
5. At school, students are rewarded (they obtain good grades, praise) for their efforts,
6. At school, children obtain the grades they deserve,
7. At school, students who obtain good grades are those who have worked hard,
8. Willingness is not always enough to succeed at school.
